# Cell Culture Adaptive Amino Acid Substitutions in FMDV Structural Proteins: A Key Mechanism for Altered Receptor Tropism

**DOI:** 10.3390/v16040512

**Published:** 2024-03-27

**Authors:** Hassan Mushtaq, Syed Salman Shah, Yusra Zarlashat, Mazhar Iqbal, Wasim Abbas

**Affiliations:** 1 Health Biotechnology Division, National Institute for Biotechnology and Genetic Engineering-C (NIBGE), Faisalabad 38000, Pakistan; hassanmushtaq245@gmail.com (H.M.); hamzamgondal@gmail.com (M.I.); 2 Pakistan Institute of Engineering and Applied Sciences (PIEAS), Islamabad 45650, Pakistan; 3 Department of Biotechnology and Genetic Engineering, Hazara University, Mansehra 21300, Pakistan; 4 Department of Biochemistry, Government College University, Faisalabad 38000, Pakistan

**Keywords:** foot-and-mouth disease virus, virus capsid, integrins, heparan sulfate, structural proteins, BHK-21

## Abstract

The foot-and-mouth disease virus is a highly contagious and economically devastating virus of cloven-hooved animals, including cattle, buffalo, sheep, and goats, causing reduced animal productivity and posing international trade restrictions. For decades, chemically inactivated vaccines have been serving as the most effective strategy for the management of foot-and-mouth disease. Inactivated vaccines are commercially produced in cell culture systems, which require successful propagation and adaptation of field isolates, demanding a high cost and laborious time. Cell culture adaptation is chiefly indebted to amino acid substitutions in surface-exposed capsid proteins, altering the necessity of RGD-dependent receptors to heparan sulfate macromolecules for virus binding. Several amino acid substations in VP1, VP2, and VP3 capsid proteins of FMDV, both at structural and functional levels, have been characterized previously. This literature review combines frequently reported amino acid substitutions in virus capsid proteins, their critical roles in virus adaptation, and functional characterization of the substitutions. Furthermore, this data can facilitate molecular virologists to develop new vaccine strains against the foot-and-mouth disease virus, revolutionizing vaccinology via reverse genetic engineering and synthetic biology.

## 1. Introduction

**Figure 1:**
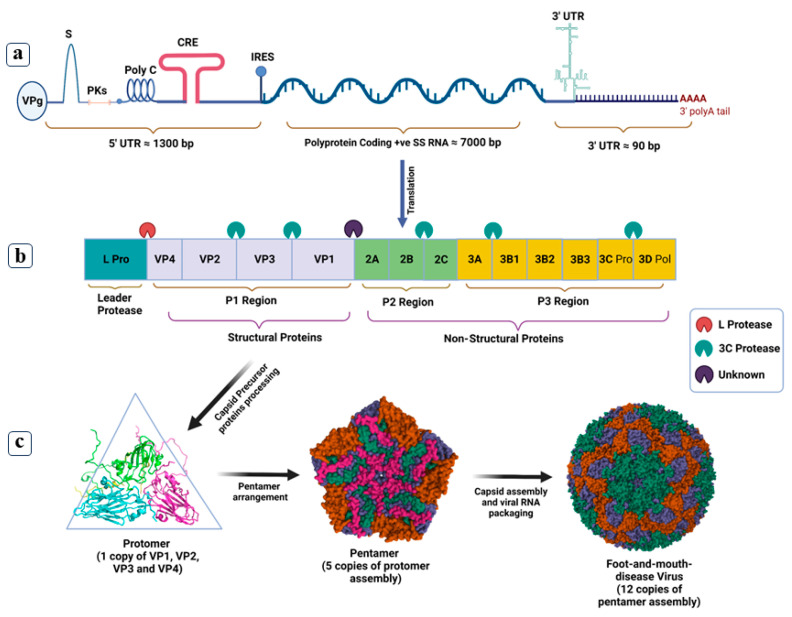
Genomic organization of FMDV and capsid assembly. Panel (**a**): The FMDV genome contains a 5′ UTR covalently attached to a viral genome linked protein (VPg) and a large open reading frame followed by a 3′ UTR with a 100 base pair long stem-loop structure. Panel (**b**): A large open reading frame encodes a polyprotein precursor processed into four distinct structural and eleven non-structural proteins via cellular and viral proteases. Non-structural proteins, including Lb pro, Lab pro, 2A, 2B, 2C, 3A, 3B1, 3B2, 3B3, and 3D, chiefly regulate the FMDV maturation and replication, while structural proteins VP1, VP2, VP3, and VP4 are building blocks of the virus capsid. Structural proteins are synthesized during the initial cleavage of the polyprotein P1 region, such as VP1, VP0, and VP3, and are assembled to form an outer capsid. The further cleavage of VP0 produces VP2 and VP4 proteins arranged to make internal capsid surfaces. Panel (**c**): The FMDV capsid has a T = psuedo3 icosahedral symmetry, tightly packed with twelve asymmetrical pentamers, each containing five protomers made up of four structural proteins.

The foot-and-mouth disease virus (FMDV) is an aphthovirus belonging to the *picornaviridae* family responsible for Foot-and-mouth disease (FMD). The FMDV is characterized as a non-enveloped, single standard, positive sense RNA virus with an approximately 8.4 Kb genome and icosahedral symmetry [1]. The FMDV genome transcribes a large open reading frame, encoding four structural and eleven non-structural proteins (Figure 1). Error-prone replication of RNA polymerase and a lack of proofreading activity have originated seven major serotypes of the FMDV: Serotype A, Asia-1, C, O, SAT1, SAT2, SAT3, and several sub-serotypes. The FMDV causes acute vesicular illness affecting over 70 animals, mainly cloven-hooved, including cows, goats, sheep, pigs, and buffalo [2]. The prevalence of global FMDV peaked in the middle of the 20th century, but timely control efforts and mitigation strategies have reduced it. The World Organization for Animal Health (WOAH) reported FMD as a top infectious disease [3] and certified 70 countries as FMD-free without vaccination. However, FMD is still endemic in Pakistan, India, and 100 other countries [4,5].

Chemically inactivated FMD vaccines have been shown to be an effective tool against the control and eradication of FMD from both enzootic and non-enzootic environments [6]. The inactivated vaccine development process is significantly hampered due to a lack of immunological cross-reactivity across FMDV serotypes and between some strains within a serotype [7]. The production of an effective inactivated polyvalent FMDV vaccine is critically dependent on the adaptation of field viruses in suspectable cell lines [8,9,10,11]. FMDV field isolates utilize four types of integrins (αVβ1, αVβ3, αVβ6, and αVβ8) as primary receptors (integrin-dependent infection), but cell culture adapted FMDVs show altered receptor tropism for the secondary receptor, Heparan sulfate (HS) [12]. FMDV adaptation is generally achieved through serial cytolytic infections imparting the propagation ability to FMDV variants in cell lines having a low or negligible expression of integrins (integrin-independent infection), i.e., CHO cells and BHK-21 cells. Surprisingly, FMDV adaptation is indebted by amino acid substitutions in viral capsid which facilitate attachments between structural proteins and heparan sulfate glycosaminoglycans, also called the heparan sulfate receptor (ubiquitously expressed linear polysaccharides on the cell surface and in the extracellular matrix) [13]. Several studies have reported that the positively charged amino acid substitutions in the exposed loops of VP1, VP2, and VP3 enable the binding of FMDV with negatively charged heparan sulfate, initiating virus internalization via caveola-mediated endocytosis [14].

The aim of this study is to summarize the current progress and scientific studies available on the adaptation of FMDVs in cell culture. Furthermore, this study attempts to highlight the structural properties of FMDV capsid proteins, the role of critical residues responsible for virus adaptation, and the functional characterization of adaptive amino acid substitutions.

## 2. Adaptive Amino Acid Substitutions in Capsid Proteins

### 2.1. VP1 Protein

#### 2.1.1. Structural Properties

**Figure 2:**
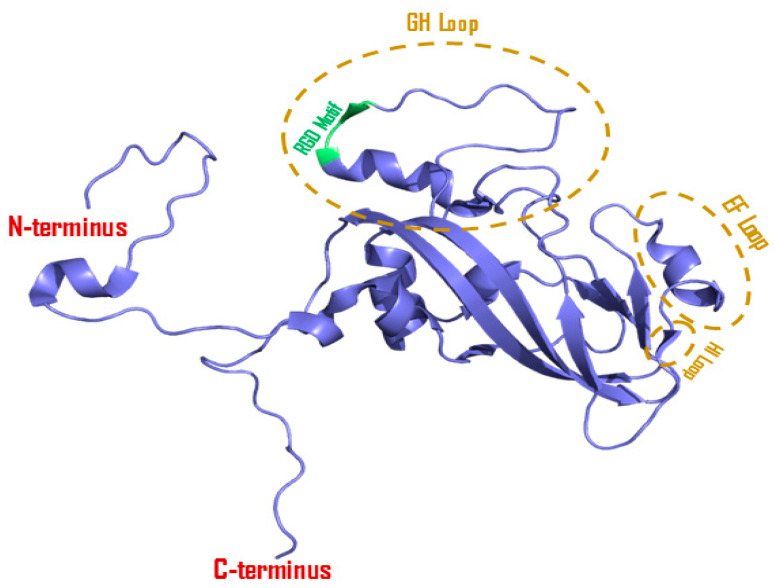
Cartoon diagram of a VP1 protein. A 3D model of VP1 was constructed from serotype O FMDV (PDB ID: 1FOD) with PyMOL protein visualization software, V2.5.4. The RGD motif that is mainly responsible for binding with integrin receptors is highlighted in green color, while surface-exposed loops (i.e., EF loops and HI loops) are highlighted in brown circles.

The protein alignment of VP1 shows 210 or 211 amino acid residues for serotype A, 209 or 210 amino acid residues for serotype Asia-1, 207 or 209 amino acid residues for serotype C, and 213 amino acid residues for serotype O. Almost 74% of the amino acid residues in VP1 are highly variable [15,16]. The inevitability of the VP1 protein relies on its three major features: defined antigenic sites, an RGD motif for receptor interactions, and a highly flexible G-H loop [17]. First, the antigenic site is mapped at the apex of the G-H loop, which contains an RGD motif at residues 144 (R), 145 (G), and 146 (D), making an open turn prior to the 310 helix (Figure 2) [14]. The RGD motif not only contributes to antigenicity but also serves as an attachment site for integrin receptors [18,19]. Molecular simulations revealed that the G-H loop naturally acquires down confirmation and protrudes from the capsid surface like a tentacle fluctuating actively for integrin binding [14,16,20]. The second antigenic site is positioned within the C-terminus of VP1, having 208 as the critical residue for antibody recognition and binding. The B-C loop of VP1 contributes to the third antigenic site to encompass critical residues at the 43–45 position, present near the five-fold axis. These antigenic sites are mostly similar in all serotypes of FMDV [15].

#### 2.1.2. Adaptive Amino Acid Substitutions in VP1

The VP1 capsid protein is the most studied structural protein in terms of adaptive amino acid substitutions. Mapping of critical adaptive amino acid substitutions in the VP1 region has employed reverse genetic approaches for the generation of site-directed mutagenic viruses as well as synthetic viruses for an effective antigen production process. The characterization of beneficial adaptive substitutions is generally based on the location of residue, frequency of substitution, nature of residue change, and implication for receptor plasticity [21]. FMDV serotype A VP1 surface exposed residues are highly prone to substitutions, including 41, 46, 83, 85, 95, 108, 110, 142, 148, 194, 195, and 210 residues as most critical for receptor tropism during cell culture adaptations [22]. These residues are mostly located near or inside the G-H loop, HS binding site, and five-fold axis symmetry, influencing receptor selectivity. Here, our literature review briefly illustrates several adaptive substitutions and their impact on receptor affinity and antigenic characteristics.

Substitution at the VP1 83 residue is considered to be a major determinant of FMDV cell culture adaptation in serotype O viruses [23]. The substitution of glutamic acid to a positively charged lysine at VP1 residue 83 (E83K), in combination with lysine to methionine at VP2 residue 80 (L80M), showed a crucial role for integrin-independent infection in serotype O viruses [24]. A recent study claimed that E83K substitution interplays with Type-1 IFN signaling to evade the host humoral immune response, resulting in the attenuation of the virus. Although pathogenicity in suckling mice was reduced, immunization induced significant levels of neutralizing antibodies. Surprisingly, E83K substitution harboring the FMDV exhibited HS receptor tropism with improved replication ability in cell culture, indirectly suggesting a new approach for virus attenuation in the vaccine production process [25].

Positive amino acid substitution at residue 95, located in five-fold axis at the junction of two VP1 proteins, is found to be critical for cell culture adaptation of serotype A viruses. Glutamic acid to Lysine replacement (E95K), alone or in combination with Serine to Leucine (S96L) upstream region of an RGD motif, serves as the interaction site of VP1 with the C-terminus of the JMJD6 protein. Recombinant viruses containing a KGE motif, in combination with E95K, potentially infected CHO cells in a non-integrin and HS-independent manner [26]. Lawrence et al. [27] further extended the study and evaluated the pathological properties of the JMJD6-FMDV (containing E95K, S96L, and KGE motifs) in cattle via inoculation through aerosols and intraepithelial injections. The JMJD6-FMDV virus showed no clinical signs and viremia in aerosol-inoculated cattle, but intraepithelial inoculation showed pathogenesis equal to a wild-type FMDV infection, suggesting the in-vivo interaction of the JMJD6-FMDV with the JMJD6 protein depends on the route of entry.

The fixation of positive amino acid residues at the 110–112 position in the βF-βG and at the 83–85 position in the βD-βE of VP1 among SAT viruses revealed adaptability against the HS receptor. Forming a positive charge through the substitution of glycine to arginine at the 112 position (G112R) introduces a KGR motif instead of a wild KGG in the pore of the heparan binding site, facilitated by the close proximity of a Q72R substitution with positively charged residues in the C-terminus of VP1, contributes to the adaptation of FMDV serotypes O, A, and Asia-1 in BHK-21 cell line [14]. Substitutions in the G-H loop of VP1 have been widely characterized for their critical role in receptor usage and antigenic properties. Serially passaged Asia-1 FMDV in suckling mice introduced the substitution of serine to aspartate at residue 154 (S154D) and resulted in increased replication and receptor tropism for αυβ8 integrins [28].

The substitution of glutamic acid with lysine at residue 202 (E202K) of the serotype A virus, located at the edge of the protomer at the VP2 and VP3 interface, showed infectivity for BHK-2P cells. Additionally, E202K substitution, along with Q110K, in VP1 (residue 110 located near the five-fold symmetry axis) or E59K in VP3 allowed mutant viruses to infect CHO-K1 cells in an integrin and HS independent manner, possibly via the JMJD6 receptor [8,20]. Berryman et al. [14] also supported the idea that Q110K substitution in serotype O and Asia-1 viruses introduces a KGD motif in place of an RDG motif and facilitates infection in HS-negative CHO cells.

Lee et al. reported the substitution of a surface-exposed proline to leucine at position 208 (P208L), located near the already identified critical residues 95–96 of VP1, introduce hydrogen bonding between VP1 leucine and JMJD6 proteins at 300 and 314 residues which facilitate JMJD6 receptor interaction [29]. Negatively charged amino acid substitution, asparagine to glutamic acid or aspartic acid, at residue 17 has been widely studied, which provides virus stability against acidic pH by incorporating 60 negative charges on the capsid surface [30]. Table 1 lists the reported amino acid substitutions in the VP1 capsid protein, along with their roles in the adaptation process.

The adaptation of field isolates in cell culture can result in a change of viral properties, i.e., virulence, pathogenicity, and antigenicity. Bai et al. [31] suggested that C275I in IRES, T142A, A152T, and Q153P in VP1, combined with E136G in VP2 and A174 S in VP3, enhanced the pathogenicity of adapted virus in 3-day-old-suckilng mice. Bøtner et al. [32] reported the attenuation of the FMDV serotype O virus in cattle during adaptation in BHK-21 cells. They proved the amino acid substitutions in capsid-exposed proteins responsible for this attenuation. Several studies have reported reduced virulence of cell culture-adapted FMDVs that gets rapidly clear from the animal body, avoiding virus spread and disease conditions. Zhao et al. [33] inoculated cell culture-adapted FMDV serotype O viruses having K at 83 and R at 172 residues of the VP1 in pigs. Their findings concluded that the fixation of positive charges on the capsid surface reduces the pathogenicity as the charged residues facilitate the clearance of viral particles, preventing the interaction of the virus with specific cell types required for lesion formation. Capsid coding sequences are considered determinants of pathogenicity in pigs. Lohse et al. [34] concluded that the cell culture-adapted OK1 B64 virus did not show disease symptoms, but the chimeric viruses O1K/A-TUR and O1K/O-UKG (having a backbone of OK1 but capsid sequences from filed isolates) exhibited strong pathological conditions in pigs. In contrast, Lian et al. [28] have suggested that S154D in the VP1 protein increases the rate of virus replication in BHK-21, PK-15, and IB-RS-2 cells and enhances pathogenicity in pigs.

**Table 1:** Reported amino acid substitutions in FMDV VP1 protein and their roles for virus adaptation.

FMDV Serotype	Amino Acid Substitutions	Loop	Role of Substitution	Reference
A	T48I	B-C loop	Confers serological heterogenicity	[22]
A	Q58K	BC-BD loop	Introduced positive charge for HS interaction during adaptation in IB-RS-2 cells	[35]
A	L130P	BG2 loop	Facilitated interaction with JMJD6 protein	[35]
A	A143V	G-H loop	Confers serological heterogenicity	[35]
A	L150P	G-H loop	Supported the usage of JMJD6 receptor	[35]
A	I154N	BG-BH loop	Juxtaposed on capsid surface, modulated receptor interaction, and antigenicity during adaptation in BHK-21 cells	[9]
A	Q157R	BG-BH loop	Facilitated adaptation of FMDV in BHK-21 suspension culture	[36]
A	E194K	BH-B1 loop	Introduced positive charge in HS-binding pocket during FMDV adaptation in IB-RS-2 cells	[37]
A	S196K/T	C-terminus	Introduced positively charged residue interacting with HS-binding pocket	[26]
A	H201R	C-terminus	Occurred during passaging virus between LFBK, BHK-21, and IB-RS-2 cells, facilitated receptor tropism for negatively charged HS GAGs	[26]
Asia-1	V86A	BB-BC loop	Facilitated BHK-21 adaptation	[26]
Asia-1	V148D	BG-BH loop	Critical for antigenicity and cell culture adaptation	[38]
C	N17D	N-terminus	Modulated virus resistance against pH	[30]
C	R97H	-	Loss of heparin binding capacity during BHK-21 adaptation	[39]
C	T148K	N-terminus	Facilitated BHK-21 adaptation	[40]
C	G194D	N-terminus	Facilitated BHK-21 adaptation	[40]
O	A13T	N-terminus	Provided capsid stability	[41]
O	Q25R	N-terminus	Confers acid stability	[42]
O	K45Q	B-C loop	Facilitated HS receptor interaction	[23]
O	Q47K	B-C loop	Present in antigenic site 3, strongly associated with neutralizing antibody binding	[43]
O	E83K	BD-BE loop	Provided selective advantage for BHK-21 adaptation, Interplays with type 1 INF-signaling for neutralization antibody production	[44]
O	N85D	B-E loop	Bovine attenuation of FMDV	[44]
O	D137G	G-H loop	Compensate deleterious effects of L80M VP2 substitution important for HS receptor tropism	[24]
O	S139R	G-H loop	Close to antigenic site 1, facilitated HS receptor interaction	[23]
O	T142N/A	G-H loop	Surface exposed residue, influences virus interaction with HS receptor	[31,45]
O	V144L	G-H loop	Critical residue for antigenic site 1	[21]
O	A152T	G-H loop	Critical residue for antigenic site 1, influence virus interaction with HS receptor	[46]
O	A155V	G-H loop	Present in antigenic site 1, associated with neutralizing antibodies binding	[43]
O	Q203R	C-terminus	Surface exposed residue, facilitated BHK-21 adaptation	[47]
O	K210Q	C-terminus	Critical for VP1/2A junction cleavage	[44]
SAT1	A69G, N110L/K	BF-BG loop	Facilitated binding of Virus with negatively charged HS molecules	[48]
SAT1	N48K, E84K	BD-BE loop	Supported clustering of positive charge around the five-fold axis symmetry for improved HS interactions	[9]
SAT1	G112R/K	BF-BG loop	Located in surface-exposed loops connecting B-sheet structures, actively interact with HS GAGs	[49]
SAT1	V179E	BF-BG loop	Facilitated HS binding	
SAT1	D181N	BF-BG loop	Supported clustering of positive charge around the five-fold axis symmetry for improved HS interactions	[9]
SAT1	K206R, K210R	C-terminus of VP1	Located in the walls of heparin binding site, facilitated HS receptor binding	[50]
SAT2	E83K	BD-BE loop	Not surface exposed, but critical for HS binding	[9]
SAT2	Q85R	BD-BE loop	Provided positive charge for five-fold axis symmetry	[51]
SAT2	P110G	BF-BG loop	Facilitated HS interaction	[50]
SAT2	E161K	BG-BH loop	Fixed positively charged residue in GH-loop, affects polarity site	[52]

### 2.2. VP2 Protein

#### 2.2.1. Structural Properties

**Figure 3:**
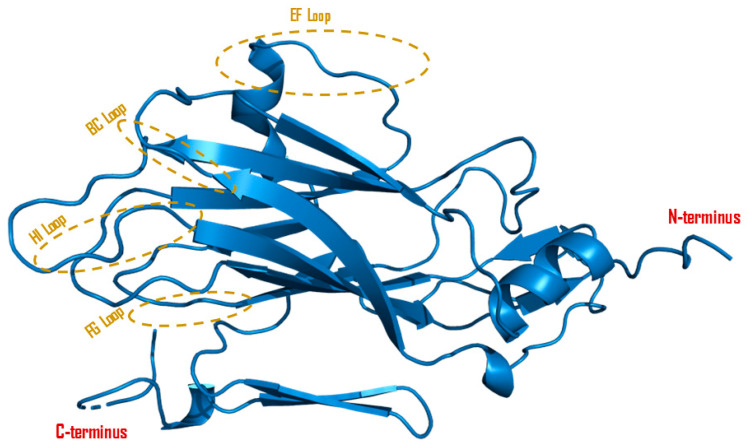
Cartoon diagram of the VP2 protein. A 3D model was constructed from Serotype O FMDV (PDB ID: 1qqp) with PyMOL protein visualization software V2.5.4. The surface exposed loops (E-F loop, B-C loop, H-I loop, and F-G loop) are highlighted in brown circles.

The protein alignment of VP2 shows 218 amino acids for Eurasian serotypes and SAT3 while showing 219 amino acid residues for SAT2 and SAT3 serotypes. VP2 is generally a conserved structural protein that plays an essential role in the maturation and stability of the capsid [53]. FMDV structural virus proteins (VPs) are wedge-shaped beta sandwiches composed of eight single strands, including C, H, E, and F exposed on the capsid surface and B, I, D, and G contributing to the internal capsid structure formation [54]. Interestingly, VP2 is responsible for antigenic variations in different serotypes of FMDV. Several studies identified conserved antigenic sites in the B-C loop (residues 70–80 located between beta-strands B and C, visible on the outer surface of virus particle) and the EF loop (residues 131–134 located between beta-strands E and F, visible on the outer surface of virus particle) of serotypes O and A, and SAT viruses (Figure 3) [55]. The VP2 protein has an influence on the virus replication and virulence properties. Xue et al. [56] elaborated by inducing substitutions in the VP2 protein, which resulted in reduced replication in BHK-21 cells and decreased virulence in suckling mice. Similarly, VP2 can boost virus replication by interacting with Heat Shock Protein Family B member 1 (HSPB1), which activates the EIF2S1-ATF4 pathway, ultimately promoting autophagy and enhanced virus replication [57]. The VP2 of the FMDV is smaller compared to the VP2 found in other picornaviruses [58,59]. VP2 N-terminal amino acids within three adjacent pentamers are organized at each three-fold symmetrical axis of the capsid to form a tightly bound C-shaped structure, which serves as a calcium-binding site [12]. Furthermore, the calcium-binding site contains a conserved glutamic acid residue at the 6th position of the protein, making a significant ionic bond for maintaining the structural stability of the viral capsid [60,61]. Crystallographic studies revealed that loops inside or outside the capsid interact with VP1, VP2, and VP3, and their C-termini consistently appear on the exterior surface while their N-termini are positioned in the inner face [62,63].

#### 2.2.2. Adaptive Amino Acid Substitutions in VP2

Various amino acid substitutions have been identified in the VP2 protein at different positions, including 65, 67, 74, 78, 79, 80, 115, 130–131, and 170, critical for altered receptor interactions [9,23,50,63,64]. Receptor tropism was identified with amino acid substitutions at positions 78–80 and 130–131 in viruses of serotypes A and O [63]. The exchanges in this region appear to change the orientation of the RGD containing the VP2 G-H loop, enabling the virus to bind to host cells using the HS or an unidentified receptor [23]. It is interesting to note that VP2 residue 121 and residue 131 are involved in the heparan sulfate binding pocket and exhibited an ability to acquire positively charged amino acids, possibly for better interaction with negatively charged HS glycosaminoglycans [23,65]. The most frequently described amino acid exchange is the substitution of the negatively charged anion glutamic acid at the position of the 131 residue with positively charged lysine (E131K), glutamic acid to lysine (E82K), and glutamic acid to glycine (E131G) in the VP2 region [36]. However, positive amino acid substitutions at residues 133, 134, and 136, located inside the HS binding depression, is another extensively reported phenomenon in serotypes A and O [35,47].

Q170H/R and T129K alteration in the SAT1 and SAT2 viruses raises the positive charge surrounding the particle’s axis. Substitution at residue 172, located in the heparan sulfate binding region, lowered the positive charge at the axis by replacing a positively charged lysine with an uncharged asparagine in the serotype A virus. These Substitutions resulted from selection pressures, other than immunological pressures, which modified the virus’s antigenic behavior [66]. K175R, a third exchange within the G-H loop of VP2 for serotype O, maintained the positive charge at this location [67]. Lysine or arginine residues have been frequently seen along with the elimination of negatively charged amino acids, and partial positive charges appear. A recent study has reported two separate examples of positively charged substitutions, E133K and Q74R, in the small depression of VP2 at the center of the three main capsid proteins for type A and O viruses [68]. Substitutions of VP2 residues 82, 88, and 131 had an effect on the capsid structure and its stability [69]. The change of amino acid composition in VP2 frequently occurs along with variations in either VP1 or VP3 [36]. The lists of reported amino acid substitutions in VP2 and their roles in virus adaptation are outlined in Table 2.

### 2.3. VP3 Protein

#### 2.3.1. Structural Properties

**Figure 4:**
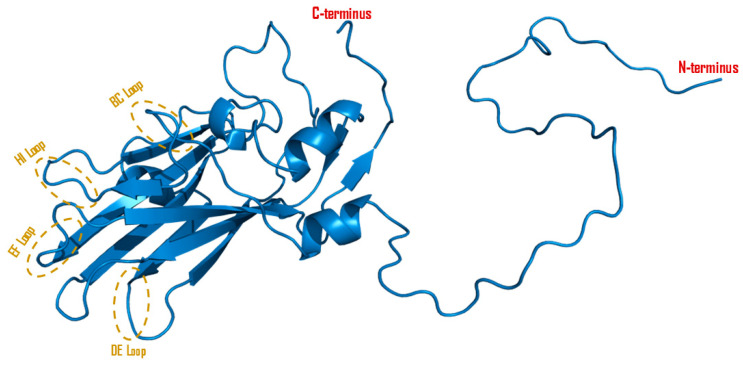
Cartoon diagram of VP3 protein. A 3D model was constructed from Serotype O FMDV (PDB ID: 1qqp) with PyMOL protein visualization software V2.5.4. The surface exposed loops (E-F loop, B-C loop, H-I loop, D-E loop) are highlighted in brown circles.

The protein alignment for VP3 displays 221 amino acid residues for SAT1 and SAT3, 220 amino acid residues for serotype O, 219 amino acid residues for serotypes Asia-1 and serotype C, and 220 and 221 amino acid residues for highly variable serotype A. Only 39% of the VP3 amino acid residues were invariant, indicating considerable variability [59]. The viral protein VP3 is essential for the generation of viral capsids and is involved in receptor binding, positioned around the three-fold axis [81]. Pentameric protomers (five copies of protomers combined, Figure 1) are connected by the N-termini of the five copies of VP3 that are twisted around the five-fold axis, contributing to the creation of an axial channel to enable the quick entry of tiny molecules like cesium ions into the particle. This N-terminus and surface exposed loops play important roles in packing protomers into pentamers, viral–host interactions, and the adaptation to different cellular environments (Figure 4) [61,82]. VP3 amino acids phenylalanine, valine, and cysteine are mostly conserved at positions 3, 5, and 7, respectively, making the pore shape extremely hydrophobic [67]. VP3 also plays a critical role in harnessing the host immune response for enhanced replication by degrading Histone deacetylase 8 (HDAC8) through AKT-mTOR-ATG5-dependent autophagy [83]. Li et al. [84] further identified the role of the C-terminus of VP3 structural proteins in evading host innate immunity. The C-terminal 111–220 amino acid residues of VP3 interact with virus-induced signaling adapter (VISA) and block IFN-β signaling pathways, supporting virus pathogenesis.

#### 2.3.2. Adaptive Amino Acid Substitutions in VP3

Sequence analysis of the VP3 capsid protein in SAT1 and SAT2 revealed modifications in nine amino acids. Only two of these, i.e., glutamic acid to lysin at residue 135 and glutamic acid to lysin at residue 175, were positively charged and situated within the surface-exposed loops of VP3 [50]. A group of substitutions around the N-terminus of VP3 for SAT viruses (T43S, Q49E) and type C (N13H, M14L, A25V) have been observed during serial passaging of the virus in BHK-21 cells [39,85]. The highly variable amino acids between residues 55 and 88 make up the HS binding site of the virion [15]. Multiple studies describe mutations towards a positive charge as the 56 position for serotypes A and O [86], including the β-knob of VP3 to produce one of the outer layers of the HS binding depression [15]. Serotype Asia-1 has been reported to exchange a negatively charged glutamate with a positively charged lysine at residue 59, which facilitated virus interactions with the negatively charged HS receptor. This residue is part of the HS binding site on the viral particle and situated in the loop downstream of the B1 strand [8].

Amino acid residues from 130 to 140 are considered highly variable regions of VP3. Substitutions towards a positive charge in the surface-exposed loops of the E-F region (T129K, E135K) appear to be valuable for SAT serotype adaptation in cell culture [50]. Two amino acid substitutions in this variable area, E138G and K139E, have been reported for serotype A viruses adapted to BHK-21 suspension cells, while a D132N substitution for SAT viruses adapted to adherent BHK-21 cells [67]. Furthermore, another “adaptive hot spot” is between positions 173 and 180, where numerous variations have been identified during the adaptation of serotypes A, O, C, and SAT1 viruses [35,39,44]. Table 3 lists all previously reported amino acid substitutions in VP3 and their roles in the adaptation process.

### 2.4. VP4 Protein

#### 2.4.1. Structural Properties

**Figure 5:**
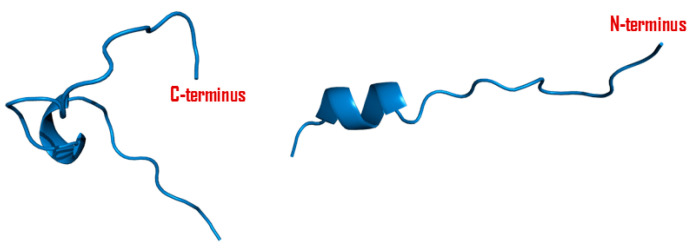
Cartoon diagram of VP4 protein. A 3D model was constructed from Serotype O FMDV (PDB ID: 1qqp) with PyMOL protein visualization software V2.5.4. The structure of VP4 residue 40 to 64 is still unresolved and shown here in the form of a gap.

VP4 is the most widely conserved FMDV protein, having only 29 variant amino acid residues with 85 total amino acid residues, and only 15–39 and 65–85 residues have been identified [31,91]. VP4 is enclosed inside the capsid along with the genomic viral RNA and appears to be primarily involved in RNA interactions [63]. This hydrophobic protein is primarily found in the interior of the capsid and plays a key part in virus entrance (Figure 5) [92]. The myristoylated N-terminus of VP4 is located near the five-fold axis symmetry, and the C-terminus is situated near the three-fold axis; making a helix of three turns facilitates the membrane permeability of the endosome [60]. A study has suggested the role of a myristoylated N-terminus in the stability of β-annulus (axial pore) is formed by the VP3 protein N-terminus [12]. VP4 is momentarily exposed at the capsid surface during a process known as capsid “breathing” [61]. VP4 also contains an MHC-promiscuous T-cell epitope at the region of 20–35 residues. This epitope site has been linked to induce an innate immune response in T-cells via recognizing the T-cell epitopes of MHC [93].

#### 2.4.2. Adaptive Amino Acid Substitutions in VP4

VP4 of the FMDV is the most conversed structural protein, and amino acid substitutions in VP4 have rarely been reported. The single substitutions for serotype A and O viruses have been reported [44], as well as along with additional amino acid substitutions in other proteins [66]. The amino acid chain of VP4 forms a three-turn helix, and the myristoylated C-terminus is close to the three-fold axis, while the N-terminus is near the five-fold axis of the particle [61]. Five copies of the VP1 subunit are positioned along the five-fold symmetry, while three copies of each VP2 and VP3 subunit alternated within the three-fold axis of the capsid, and the VP4 subunit is located internally [77]. Studies have identified negligible adaptive substitutions in VP4, which were identified but not functionally characterized, which limits the comprehensive understanding of their effects on the virus. Table 4 list previously reported amino acid substitutions in VP4. 

## 3. Conclusions

The foot-and-mouth disease virus has been considered the top infectious animal virus, and researchers have prescribed several preventive measures for the control of FMD outbreaks. The adaptation of FMDV field isolates in cell culture provides significant advantages in virus attenuation, pathogenicity modulation, and antigen production for inactivated vaccine formulations. Field isolates bind with the cellular surface via RGD-dependent primary integrin receptors. Conversely, cell culture-adapted viruses bind via an altered receptor, the HS receptor. During the adaptation process, field isolates introduce amino acid substitutions in the surface-exposed loops of VP1, VP3, and VP3 proteins. Studies have characterized the VP1 protein as a major regulator of viral adaptation as it is the highest exposed protein on the FMDV capsid. Likewise, studies have extensively reported substitutions in VP1 loops, especially around the RGD motif. VP2 and VP3 proteins are responsible for the maturation and stability of capsid. However, studies have also reported considerable substitutions in VP2 and VP3, either in combination with VP1 or alone.

The adaptation process is considered the gold standard approach for high antigen yield at an affordable cost, but it is a laborious and time-consuming process, rendering the disease control programs ineffective. To overcome these limitations, knowledge of frequent amino acid substitutions and reverse genetic engineering can be employed to design recombinant viruses as vaccine seed strains. Fighting against emerging variants of FMDV poses a quick adaptation of field isolates and a huge amount of antigen production that can be compensated by introducing previously identified substitutions through site-directed mutagenesis or synthetic approaches. This developmental revolution has the potential to begin a new era of recombinant FMDV viruses exhibiting high immunogenicity and broad cross-protection. Although reverse genetic engineering can revolutionize the virus adaptation process in less time and cost, it also has limitations, i.e., unintended genetic modifications, threats of substitution reversion, ineffective immune responses due to antigenic variations, and challenges in the effective delivery of infectious clones. Furthermore, the development of recombinant FMDVs poses several ethical and biosafety concerns, including the accidental release of viruses, which significantly limits the approach.

The structural and functional characterization of adaptive substitutions is necessary to understand the patterns of substitution introduction and the critical roles in capsid stability as well as viral replication. In conclusion, this literature review emphasizes the exploration of adaptive amino acid substitutions in FMDV structural proteins for virus pathogenesis, antigenic determination, and receptor tropism. Furthermore, these substitutions can be studied in terms of capsid stability against different pH and temperature fluctuations to manage the cold-chain process.

## Figures and Tables

**Table 2:** Reported amino acid substitutions in FMDV VP2 protein and their roles for virus adaptation.

FMDV Serotype	Amino Acid Substitutions	Loop	Role of Substitution	Reference
A	F67L, H77R, Y79H	B-C loop	Protected the viral DNA and promote the viral entry into host cells	[44]
A	E79A/G	βE-βF loop	Facilitated viral interaction with host cells	[66]
A	E82A/K	βC loop	High immunogenicity, Part of heparan sulfate binding pocket	[44]
A	E131K/G	E-F loop	Modified cell binding property, residue 131 in VP2 is a component of the heparan sulfate- binding pocket	[36]
A	E134K	E-F loop	Improved binding with HS receptor	[24]
A	E79A/G, T134P	βE-βF loop	Altered structural configuration of VP1	[9]
A	L66F, K80E	-	Makes FMDV unrecognizable to the antibodies	[70]
A	W129R	βE-βF loop	Improved interaction among capsid proteins	[71]
A	N166D, Q146E	-	Altered capsid stability	[9]
A	T154M	-	Altered the structural arrangement of VP1	[9]
A	K172N	βG-βH loop	Stabilized RGD-dependent integrin interaction	[35]
A, O	C78Y, S131P	G-H loop	Part of the antigenic site, altered the ability of the virus to bind with integrins	[33,35,72]
O	Y79H, L80Q	B-C loop	Facilitated interaction with HS receptor	[23]
O	G36E	E-F loop	Facilitated interaction with HS receptor	[23]
O	D133N	-	Stabilized FMDV promoter	[73,74]
O	Y98F	G-H loop	Increased thermostability	[75]
O	A70V/G, S74P, N134E/K, V154M, T191N	-	Strongly influence the binding of neutralizing antigenic site	[76]
O	E136G	E-F loop	Influence interaction of the virus with cellular receptor	[31]
O	V132I	G-H loop	Promoted the binding of RGD motif with integrin molecules	[33]
O	F214L	E-F loop	Improved binding with HS receptor	[31]
O	R65H	B-B knob	survival of the virus via persistent infection	[23]
O	H65R	B-B knob	Involved in HS interaction	[23]
O	K175R	G-H loop	Introduced positive charge near the binding site	[67]
O	S93H/F/Y, S93C/Q/H/W, L94V, S97I/V/Q	G-H loop	Improved thermostability of capsid	[77,78]
C	A192T, G193S	E-F loop	Influence the viral ability to evade the host immune system	[40]
C	A277T/V	-	Influence on antigenic variation and immunological response	[79]
SAT1	Q170H, S196N	B-C loop	Enhanced the ability of the virus to stick to negatively charged sulfated polysaccharides	
SAT1	Q74R, E133K, D134E, D39A, E133K, L115Q, V90I, A107V	B-C loop	Improved SAT1 capsid interactions with HS receptor	[68]
SAT2	I32V, T43S, Q49E, M77T, E96Q, Q170R	G-H loop	Altered VP2 protein antigenic properties	
SAT2	T99A, K128E, L147F, T158I, F191L	C-D loop	Influenced interaction with HS receptor and facilitated viral entry	[68]
SAT3				
SAT1, SAT2	Q170H, R	Q170 H in B-C loop of SAT1, Q170R in G-H loop of SAT 2	Promoted viral attachment and cellular entry	
Asia-1	D72N	B-C loop	No response to monoclonal antibodies	[56]
Asia-1	H145Y	H-I loop	Provided resistance against pH	[80]

**Table 3:** Reported amino acid substitutions in FMDV VP3 protein and their roles for virus adaptation.

FMDV Serotype	Amino Acid Substitutions	Loop	Role of Substitution	Reference
A	P4S	-	Improved viral replication	[44]
A	C56R	β-B knob	supported tropism for HS receptor	[37]
A	E58K/G	B-B knob	-	[87]
A	H85Q/R	βC-βD loop	Did not facilitate HS receptor interaction	[9,37]
A	M86V/T	-	Critical residue for HS binding pocket	[9]
A	E131K/N/G/D/A/H	E-F loop	May act as mediators for receptor tropism	[35,87]
A	E138G, K139E	E-F loop	Improved modifications within the HS binding pocket	[9]
A	D174G, V174A, T174K	βG-βH loop	Facilitated interaction with HS receptor during BHK-21 adaptation	[35]
A	E177A	G-H loop	Altered configuration on binding with M8 monoclonal antibody	[88]
A	K76E	-	Critical residue located within the wall of the HS binding pocket	[35]
O	H56R	β-B knob	Introduced positive charge facilitating HS binding	[35]
C	C7V, D9A, N13H, M14L	-	Introduced disulfide bridges on the five-fold axis of capsid	[40]
C	A25V, Q218K	G-H loop	Causes significant antigenic variation	[89]
C	E173K	-	Increased viral–host tropism	[90]
SAT1	E135K, E175K, D09V	E-F loop	Responsible for developing the ability to interact with HS ionically	[68]
SAT1	A180V	G-H loop	Attaining the smooth virion appearance	[50]
SAT1	N192Y, S217I	N192Y in H-I loop, S217I on C-terminus	Facilitated HS receptor binding	[48]
SAT2	S203T, S219L, R220H	G-H loop	Facilitated HS receptor binding	[68]
SAT2	T43S, Q49E, T129K, D132N, E148K, P192T	E-F loop	-	[50,67]
Asia-1	E59K	-	Critical for heparin binding site	[8]

**Table 4:** Reported amino acid substitutions in FMDV VP4 protein.

FMDV Serotype	Amino Acid Substitutions	Loop	Reference
A	S15A/F/I	N-terminus	[94]
A	L71P	-	[70]
A	T48C/S77G/A229G	-	[66]
O	A8S	N-terminus	[23]
O	T52A	-	[44]

## Data Availability

Not applicable.
